# The Impact of Multileaf Collimator Size on Single Isocenter Dynamic Conformal Arcs-Based Radiosurgery for Brain Metastases

**DOI:** 10.7759/cureus.58816

**Published:** 2024-04-23

**Authors:** Yoshiko Oshiro, Yuichi Kato, Masashi Mizumoto, Hideyuki Sakurai

**Affiliations:** 1 Radiation Oncology, Tsukuba Medical Center Hospital, Tsukuba, JPN; 2 Radiation Oncology, University of Tsukuba Hospital, Tsukuba, JPN

**Keywords:** mbm, brain, stereotactic radiotherapy, mlc, single isocenter, brain metastases, srs, radiotherapy, radiosurgery, multileaf collimator

## Abstract

Purpose: To compare the plan quality of stereotactic radiosurgery (SRS) between 2.5-mm and 5-mm multileaf collimator (MLC) and investigate the factors’ influence on the differences by MLC size.

Methods: Seventy-six treatment plans including 145 targets calculated with a single isocenter multiple noncoplanar dynamic conformal arc (DCA) technique using automatic multiple brain metastases (MBM) treatment planning system. Conformity index (CI), gradient index (GI), lesion underdosage volume factor (LUF), healthy tissue overdose volume factor (HTOF), geometric conformity index (g), and mean dose to normal organs were compared between 2.5-mm and 5-mm MLC. Then the factors that influenced the differences of these parameters were investigated. The impact of target size was also investigated for CI and GI values of individual targets (n=145), and differences between 2.5-mm and 5-mm MLC were analyzed.

Results: All parameters except for LUF were significantly better in plans with 2.5 mm MLC. Target size was a significant factor for difference in HTOF, and distance between targets was a significant factor for difference in brain dose and GI. Among 145 metastases, the average inverse CI was 1.35 and 1.47 with 2.5-mm and 5-mm MLC, respectively (p<0.001). The average GI was 3.21 and 3.53, respectively (p<0.001). For individual targets, target size was a significant factor in CI and GI both with 2.5-mm and 5-mm MLC (p-value: <0.001, each). CI and GI were significantly better with 2.5-mm than 5-mm MLC. CI was almost >0.67 except for ≤5mm targets with 5-mm MLC. Also, GI was almost smaller than 3.0 for >10 mm targets both with 2.5-mm and 5-mm MLC.

Conclusions: MBM with 5-mm MLC was almost fine. However, it may be better to use a conservative margin for larger metastases. It may also be better to avoid SRS with 5-mm MLC for patients with ≤5 mm target size.

## Introduction

Stereotactic radiosurgery (SRS) is increasingly used for brain metastases [1-3] due to improved technology and emergence of single-isocenter SRS solutions in recent years. The dosimetric results of linac-based SRS are comparable to those with cyberknife (CK) and gamma-knife [4-6] and multiple brain metastases (MBM) as small as a few millimeters can be targeted. A multileaf collimator (MLC) design has also been developed, with production of an MLC with a resolution of 2.5 mm (HD 120, Varian Medical Systems, Palo Alto, CA, USA). However, since the total number of leaves remains the same, the size of the maximum irradiation field becomes smaller when a 2.5-mm MLC is used (maximum field size: 40×40 cm vs. 22×40 cm). This is of no concern when the machine is used for dedicated high-precision radiotherapy because a fine MLC can better fit the shape of the metastases. However, in facilities with only 1-2 linacs, which is often the case in Japan, the maximum irradiation field size becomes an issue because versatility is required.

Several studies have compared 2.5-mm and 5-mm MLCs [7-11]. The findings have indicated some improvements in the dosimetry parameters by switching from a 5-mm to a 2.5-mm MLC, but the degree to which this is clinically significant is unclear. This raises the question of whether it is possible to conduct SRS for small metastases of a few millimeters with a 5-mm MLC. In this study, we examined specific differences in plan quality between 2.5-mm and 5-mm MLCs according to tumor status (size, number, and tumor location) and investigated the feasibility of SRS for small metastases using the MBM technique with a 5-mm MLC.

## Materials and methods

Treatment plans

The study included 76 plans for 145 targets in 46 patients treated with CK. These plans were used in a previous study using a single isocenter multiple noncoplanar dynamic conformal arc (DCA) technique on an Elements ver. 4.0 automatic MBM planning system (Brainlab AG, Munich, Germany) equipped with a 2.5-mm MLC (HD 120, Varian Medical Systems, Palo Alto, CA, USA) [6]. The plans were further replanned with a 5-mm MLC (Millenium, Varian Medical Systems) on Elements MBM ver. 4.0. Among the plans, 44 and 32 included single and multiple metastases, respectively; the mean target size ranged from 4.2 to 65.6 mm; and 24 plans included irregularly shaped targets. The plan details are shown in Table 1. For plans including multiple targets, the mean distance between the targets ranged from 9.9 to 96.8 mm (median, 66.4 mm).

**Table 1:** Details of treatment plans (n=76)

Item		Number of plans
Target number	Single	44
(Range: 1-16)	Multiple	32
Mean target size (mm)	<5	2
Median: 20.0 mm	5-10	11
Range: 4.2-65.6 mm	10-20	25
	20-30	19
	≥30	19
Target shape	Circular	52
	Irregular	24
Distance from brainstem (mm)	Inside	4
	<5	15
	5-10	3
	10-20	4
	20-30	3
	≥30	47

The treatment beams were equipped with flattening filter free 6 MV photon beams. The number of arcs was determined automatically depending on the spatial distribution of the metastases. The arc consisted of at least one coplanar arc and three noncoplanar arcs with couch angles of 0, 45, 135, and 270°. Pencil Beam Convolution was used for inverse planning with a dose grid resolution of 2.0 mm. The treatment plans were automatically adapted based on the clinical treatment protocol. The treatment plans using 2.5-mm and 5-mm MLCs were then compared.

Data analysis and dosimetric parameters

Whole Plan Quality

The mean dose to the brain and the following dosimetric parameters were calculated from the dose volume histogram (DVH) of the target volume (TV), brain, and external tissues. The Paddick conformity index (CI) [12] and gradient index (GI) [13] were calculated as follows:

CI = (TVpiv)^2^/(TV × PIV),

GI = PIV_50_/PIV,

where PIV is the prescription isodose volume, and TVpiv is PIV within the target volume.

The lesion underdosage volume factor (LUF), which is the percentage of the TV not covered by the prescription isodose [14]; the healthy tissue overdose volume factor (HTOF), the percentage of healthy tissue volume outside the TV receiving more than the prescription isodose; and the geometric conformity index (g), the sum of LUF and HTOF [14], were calculated as follows:

LUF = TV_<PI_/TV,

HTOF = HTV_>PI_/TV,

g = LUF+HTOF,

where TV_<PI _and HTV_>PI_ are the target volumes not covered by the prescription isodose, and the healthy tissue volume covered by the dose above the prescription dose, respectively. LUF, HTOF, and g provide more of a focus on the 100% prescription dose than CI or GI.

The ratios of 5-mm to 2.5-mm MLC plans were calculated to evaluate differences between the two modalities, and factors that influenced the differences, including number of targets, target shape, target maximum size, distance between targets, and distance from the brainstem, were investigated using multivariate analysis. The distance between targets was measured from the center of each target.

Plan Quality for Individual Targets

Since there were only two plans with small (≤5 mm) mean target diameters, we analyzed individual targets to investigate the impact of tumor size further. Inverse Paddick CI and GI values of each target (n=145) were collected from the plan reports for 2.5-mm and 5-mm MLCs, and these values were then compared by target size (maximum size ≤5, 5-10, 10-20, 20-30, and >30 mm). Inverse Paddick CI was calculated as:

Inverse Paddick CI = (TV×PIV)/(TVpiv),

Paddick CI =1/Inverse Paddick CI

For comparison with previous studies, Paddick CI was calculated from the Inverse Paddick CI as the inverse of the value. The impact of each target size on GI and CI was evaluated by the Kruskal-Wallis test, and differences between 2.5-mm and 5-mm MLCs were analyzed by the Wilcoxon test.

Statistical analysis

All statistical analyses were performed in SPSS ver. 27 (IBM Co., New York, NY, USA). All tests were 2-tailed and p<0.05 was considered to be statistically significant.

Ethics approval

The study was approved by the institutional review board.

## Results

All plans (n=76)

The results for all plans (n=76) are shown in Table 2. All parameters except for LUF were better in plans with a 2.5-mm MLC. The 2.5-mm to 5-mm ratios were 1.06, 0.93, 1.06, 1.00, 1.50, and 1.42 for mean brain dose, CI, GI, LUF, HTOF, and g, respectively. Differences in MLC size in HTOF were positively influenced by target size. Distance between targets negatively influenced the differences in mean brain dose and GI. These results suggest that the use of a 5-mm MLC for larger targets tended to cause overflow in the high dose area compared to the use of a 2.5-mm MLC, and the closer the distance between targets, the higher the brain-averaged dose and the larger the GI in 5-mm MLC.

**Table 2:** Dosimetric parameters for all plans IQR: interquartile range; CI: conformity index; GI: gradient index; LUF: lesion underdosage volume factor; HTOF: healthy tissue overdose volume factor; g: geometric conformity index *Italic: Negative correlation (ex. Large difference in mean brain dose for small difference between targets)

	2.5 mm MLC		5 mm MLC		p-value	5 mm/2.5 mm Median (Range)	Impact Factor on Differences By MLC Size: P-value				
	Median	IQR	Median	IQR			Target number	Target shape	Target size	Distance from brainstem	Distance between targets
Mean brain dose	109	43-194	120	52-197	<0.001	1.06 (0.01-1.81)	0.720	0.069	0.071	0.176	0.047*
CI	0.84	0.76-0.90	0.76	0.69-0.83	<0.001	0.93 (0.60-2.29)	0.900	0.652	0.083	0.511	0.643
GI	2.79	2.42-3.15	3.06	2.53-3.40	<0.001	1.06 (0.80-4.72)	0.982	0.433	0.471	0.475	0.042*
LUF	0.04	0.03-0.04	0.03	0.02-0.05	0.105	1.00 (0.10-6.00)	-	-	-	-	-
HTOF	0.17	0.11-0.31	0.32	0.21-0.45	<0.001	1.50 (0.42-23.9)	0.184	0.192	0.044	0.226	0.326
g	0.22	0.14-0.37	0.37	0.23-0.50	<0.001	1.42 (0.42-13.0)	0.304	0.215	0.087	0.279	0.500

Single target plans (n=44)

All parameters were significantly better in plans with a 2.5-mm MLC (Table 3). For a single target, there was a significant difference in LUF. Target size was a significant negative factor for differences in GI and g. Thus, a 2.5-mm MLC had greater advantages for a smaller single target.

**Table 3:** Dosimetric parameters for a single target plans IQR: interquartile range; CI: conformity index; GI: gradient index; LUF: lesion underdosage volume factor; HTOF: healthy tissue overdose volume factor; g: geometric conformity index *Italic: Negative correlation (ex. Large difference in GI for small target)

	2.5 mm		5 mm		p-value	5 mm/2.5 mm Median (Range)	Impact Factor on Differences By MLC Size: P-value		
	Median	IQR	Median	IQR			Target shape	Target size	Distance from brainstem
Mean brain dose	115	36-175	123	36-199	<0.001	1.05 (0.01-1.81)	0.339	0.950	0.621
CI	0.87	0.79-0.90	0.80	0.69-0.85	<0.001	0.93 (0.60-2.29)	0.309	0.217	0.943
GI	2.96	2.37-2.96	2.68	2.42-3.27	<0.001	1.04 (0.80-4.72)	0.526	0.011*	0.775
LUF	0.04	0.02-0.04	0.03	0.02-0.04	0.005	0.99 (0.41-3.63)	0.564	0.125	0.868
HTOF	0.14	0.09-0.29	0.26	0.19-0.56	<0.001	1.59 (0.42-23.9)	0.567	0.472	0.633
g	0.27	0.13-0.34	0.29	0.22-0.60	<0.001	1.47 (0.42-13.0)	0.914	0.024*	0.984

Multiple target plans (n=32)

The 2.5-mm plans gave significantly better results than the 5-mm plans, except for LUF (Table 4).

**Table 4:** Dosimetric parameters for multiple target plans IQR: interquartile range; CI: conformity index; GI: gradient index; LUF: lesion underdosage volume factor; HTOF: healthy tissue overdose volume factor; g: geometric conformity index *Italic: Negative correlation (ex. Large difference in mean brain dose for small distance between targets)

	2.5 mm MLC		5 mm MLC		p	5 mm/2.5 mm Median (Range)	Impact Factor on Differences By MLC Size: P-value				
	Median	IQR	Median	IQR			Target number	Target shape	Target size	Distance from brainstem	Distance between targets
Mean brain dose	97	57-209	111	60-220	<0.001	1.10 (1.00-1.29)	0.706	0.067	0.065*	0.177	0.038*
CI	0.8	0.74-0.85	0.72	0.68-0.79	<0.001	0.91 (0.69-1.52)	0.892	0.635	0.076	0.489	0.654
GI	3.06	2.67-3.51	3.33	2.93-3.79	<0.001	1.10 (0.95-1.87)	0.978	0.430	0.461	0.475	0.036*
LUF	0.04	0.03-0.04	0.03	0.03-0.06	0.854	1.00 (0.10-6.00)	-	-	-	-	-
HTOF	0.25	0.15-0.34	0.37	0.24-0.44	<0.001	1.45 (0.57-4.55)	0.173	0.194	0.041	0.232	0.292
g	0.31	0.21-0.38	0.42	0.28-0.48	<0.001	1.34 (0.67-3.23)	0.290	0.219	0.084	0.289	0.457

Distance between targets was a significant factor of differences in brain dose and GI, and target size was a significant factor for differences in HTOF.

CI and GI for individual target

**Figure 1:**
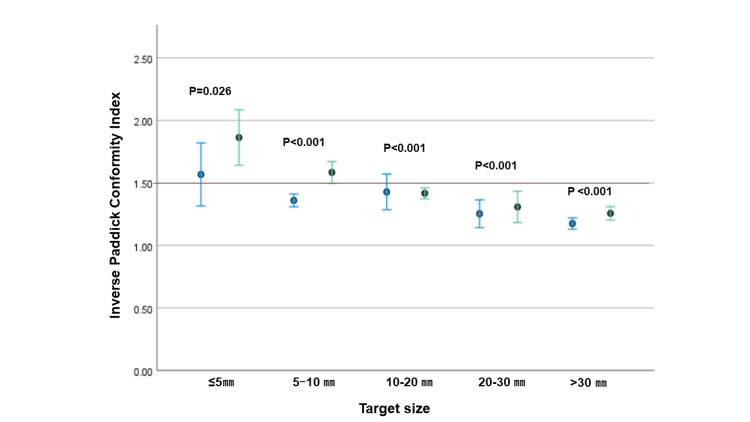
Difference of Inverse Paddick Conformity Index Difference of Inverse Paddick Conformity Index by target size. Error bars indicate 95% confidence intervals.

**Figure 2:**
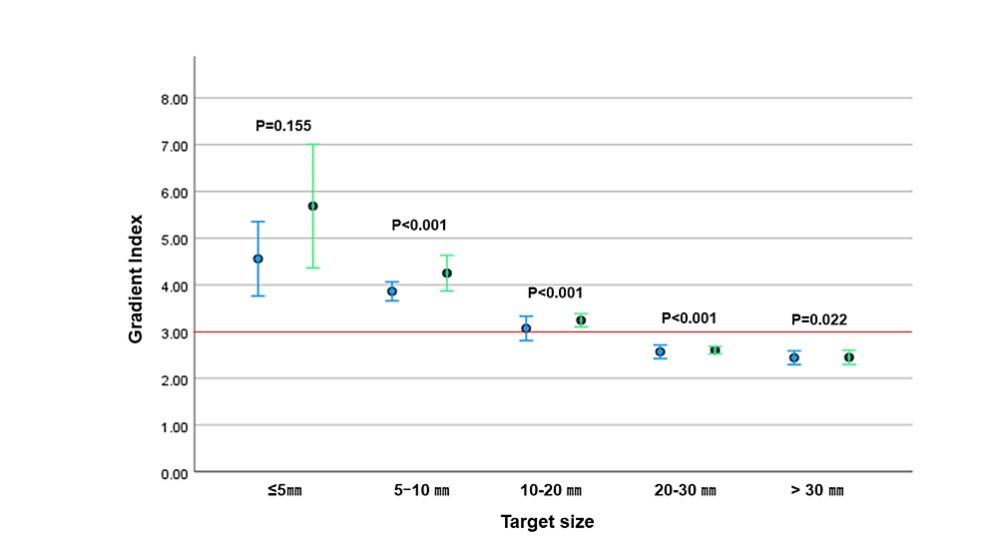
Difference of Gradient Index Difference of Gradient Index by metastatic target size. Error bars indicate 95% confidence intervals.

CI was available for 137 (94%) and 134 (92%) targets and GI was available for 116 (80%) and 110 (76%) targets with 2.5-mm and 5-mm MLCs, respectively (Table 5). The average inverse CI was 1.35 (95% confidence interval: 1.28-1.42) and 1.47 (1.41-1.53) for 2.5-mm and 5-mm plans, respectively (p<0.001), and GI was 3.21 (3.04-3.38) and 3.53 (3.29-3.77), respectively (p<0.001). Target size was a significant factor for both CI and GI with 2.5-mm and 5-mm MLCs (both p<0.001 by Kruskal-Wallis test). As the target size increased, inverse CI approached 1.0 and GI decreased. Differences of CI and GI between 2.5-mm and 5-mm MLCs are shown in Figure 1 and Figure 2, respectively. Though CI and GI were significantly better for 2.5-mm than 5-mm MLCs at almost all target sizes, the differences were particularly large in targets <10 mm. Inverse CI was mostly <1.5 (CI: >0.67) except for ≤5-mm targets with a 5-mm MLC, and GI was mostly <3.0 for >10-mm targets with both 2.5-mm and 5-mm MLCs.

**Table 5:** CI and GI for individual target CI: conformity index; GI: gradient index

Target size (mm)	Inverse Paddic CI (Paddic CI)			GI		
	2.5 mm	5 mm	p	2.5 mm	5 mm	p
All	1.35 (0.74)	1.47 (0.68)	<0.001	3.21	3.53	<0.001
≤ 5	1.57 (0.63)	1.86 (0.53)	0.026	4.56	5.69	0.155
5-10	1.36 (0.74)	1.58 (0.63)	<0.001	3.86	4.25	<0.001
10-20	1.43 (0.70)	1.41 (0.71)	<0.001	3.06	3.24	<0.001
20-30	1.25 (0.80)	1.31 (0.76)	<0.001	2.57	2.6	<0.001
>30	1.18 (0.85)	1.26 (0.79)	<0.001	2.44	2.45	0.022

## Discussion

Recent advances in diagnostic imaging have made it possible to detect small brain metastases, including MBM of a few millimeters in size [15], but it is often difficult to decide when and how to treat these lesions. SRS techniques have also improved in recent years, including development of the 2.5-mm MLC. Several previous reports have suggested an improved plan quality using a fine MLC [7-11]. Taylor et al. showed improvement in CI, GI, and the volume of normal brain receiving 12 Gy (V12) using a 2.5-mm MLC [10]. It is reasonable that a 2.5-mm MLC can provide more precise SRS, especially for small targets. However, in practice, few studies have identified the cases for which a 2.5-mm MLC is more useful. Abisheva et al. suggested that a 5-mm MLC could achieve a similar CI to that with a 2.5-mm MLC [7]. Therefore, in this study, we evaluated differences in plan quality between 2.5-mm and 5-mm MLCs and the factors that had an impact on the differences of the plan qualities, then we assessed the feasibility of using a 5-mm MLC in MBM including small targets.

Our results suggest that a 2.5-mm MLC gives significantly better plan quality and a lower mean brain dose regardless of the number of metastases. In all plans, brain dose, CI, and GI were better with a 2.5-mm MLC, indicating higher plan quality. Also, higher doses had less overflow with a 2.5-mm MLC (HTOF: 5 mm > 2.5 mm). Target size also influenced the differences in GI, HTOF, and g. The difference in GI was larger for smaller targets in single plans, which indicates that a 2.5-mm MLC is better for a single small target, whereas a larger tumor had a large impact on the difference in HTOF and g, which suggests that there is a larger high-dose overflow with 5-mm MLCs. Also, the distance between targets had a significant influence on the difference in brain dose and GI. The smaller the distance between tumors, the larger the difference in GI and brain dose. This was expected because the 2.5-mm MLC could be inserted into a small space between tumors.

Paddick and Lippitz suggested that GI should be <3 [16] and Aiyama et al. found that CI >0.65 was correlated with good disease control in SRS for single brain metastases [17]. The inverse value of 0.65 was calculated as 1.54. The current study gave median GIs of treatment plans with 2.5-mm and 5-mm MLCs of 2.79 and 3.06, respectively, and median CIs of 0.84 and 0.74, respectively. These results suggest that MBM plans were fine with 2.5-mm and 5-mm MLCs, given that our treatment plans used a single isocenter DCA technique and included tumors far from the isocenter. In addition, there was little significant difference in LUF, and it is possible to cover the TV with a 100% dose with a 5-mm MLC. Focusing on individual targets, CI was >0.65 (inverse CI <1.54) in almost all targets with a 2.5-mm MLC. However, with a 5-mm MLC, CI >0.65 was rarely achieved in targets of ≤5 mm. It was difficult to achieve GI<3 for the target of <10 mm both with 2.5- and 5-mm MLC. This was probably because the doses to each target interfered with each other in plans that included multiple targets. Differences in CI between 2.5-mm and 5-mm MLCs were significant in targets of all sizes, and differences in GI were also significant except for ≤5-mm targets.

Fine plan quality was achieved for targets >5 mm even with a 5-mm MLC. However, it seems better to set a small margin for large metastases since the prescription dose tends to overhang beyond the target (large HTOF). In MBM, larger TV margins are required for targets far from the isocenter. Therefore, it may be better to divide the isocenter into two, and it may also be better to avoid SRS for large targets adjacent to critical organs such as the brainstem and chiasma, in case these targets are far from the isocenter because there was a tendency for the dose overhang to increase. For very small metastases with a target maximum size of ≤5 mm, it seems better to avoid SRS with a 5-mm MLC, given the low CI. Metastases ≤5 mm even with margins should be treated at a facility with a 2.5-mm MLC or CK or treatment should be delayed until the lesion becomes a little larger. In addition to the current results, there may be the risk of inaccurate validation for small targets.

A limitation of the study is that all plans were not for clinical practice and not all were suitable for clinical use. However, our results provide insights into the differences and extent of these differences in treating brain metastases with a 5-mm MLC versus a 2.5-mm MLC.

## Conclusions

We conclude that MBM with a 5-mm MLC is almost fine, but that it may be better to use a conservative margin for larger metastases. It may also be better to avoid SRS with a 5-mm MLC for patients with a target maximum size ≤5 mm.
